# PROXIMA-1 beamline for macromolecular crystallography measurements at Synchrotron SOLEIL

**DOI:** 10.1107/S1600577521002605

**Published:** 2021-04-29

**Authors:** Leonard M. G. Chavas, Patrick Gourhant, Beatriz G. Guimaraes, Tatiana Isabet, Pierre Legrand, Robin Lener, Pierre Montaville, Serena Sirigu, Andrew Thompson

**Affiliations:** a Synchrotron SOLEIL, 91192 Gif-sur-Yvette, France; b Nagoya University, Nagoya 464-8603, Japan; c Carlos Chagas Institute, Curitiba, Parana, Brazil

**Keywords:** macromolecular crystallography, multiwavelength anomalous diffraction, remote access, structural biology, PROXIMA-1

## Abstract

The undulator beamline PROXIMA-1 at Synchrotron SOLEIL scheduled its first users in March 2008. The endstation is dedicated to biomolecular crystallography experiments, with a layout designed to favour anomalous data recording and studies of crystals with large cell dimensions. In 12 years, the beamline has accommodated 4267 shifts of 8 h and more than 6300 visitors. By the end of 2020, it saw 1039 identified published scientific papers referring to 1415 coordinates deposited in the Protein Data Bank. The current paper describes the PROXIMA-1 beamline, including the recent specific implementations developed for the sample environment. The setup installed in the experimental station contains numerous beam-shaping equipment, a chi-geometry three-axis goniometer, a single-photon-counting pixel-array X-ray detector, combined with a medium-throughput sample exchange robot. As part of a standard experimental scheme, PROXIMA-1 can also be accessed via ‘mail-in’ services or remotely.

## Abstract 2

PROXIMA-1 at Synchrotron SOLEIL has been run for 12 years as an endstation dedicated to macromolecular crystallography experiments, optimized for anomalous data recording and diffraction studies of large unit-cell crystals.

## 1. Introduction   

Synchrotron SOLEIL is a 2.75 GeV French synchrotron radiation facility located on the Plateau de Saclay campus in the Paris suburbs. As of 2020, Synchrotron SOLEIL can accommodate 29 endstations, among which two beamlines are dedicated to macromolecular crystallography (MX) experiments. In its early design, PROXIMA-1 was constructed around a user case for MX beamlines identified within the French community between 2000 and 2002. The beamline construction and the following improvements were executed while referring to a dedicated committee for MX studies. PROXIMA-1 observed its first beam at the sample position towards the end of 2007 and scheduled its first users in March 2008. The main purpose of PROXIMA-1, in the context of MX science, focuses on diffraction studies of large macromolecular assemblies with large lattices due to the low horizontal divergence of the beam at the sample. The introduction of a three-rotation axis goniometer in the original design of the beamline was strategic in order to optimize the diffraction experiments, which is especially important when working with large molecules. At present, PROXIMA-1 serves academic research groups located in France (55%), Europe (38%) and worldwide.

## 2. Beamline overview   

**Figure 1:**
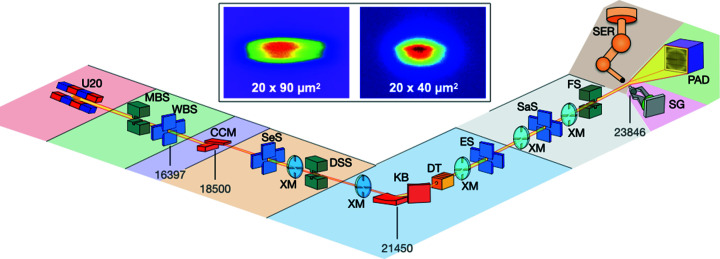
General layout of PROXIMA-1. Relative distances (not to scale) are from the light source down to the sample. U20, *in vacuum* undulator; MBS, main beam shutter; WBS, white-beam slits; CCM, channel-cut monochromator; SeS, secondary slits; DSS, downstream safety shutter; KB, KB mirrors; DT, diagnostic tools; ES, experimental slits; SaS, sample slits; FS, fast shutter; SG, SmarGon; PAD, pixel-array detector; SER, sample exchange robot; XM, X-ray beam positioning monitor. Elements are colour-located by functional blocks. The top inlet represents the direct beam at the sample position scaled and imaged on a motorized imager, with (right) and without (left) a diaphragm.

PROXIMA-1 is capable of satisfying the requirements for challenging crystallographic experiments, such as anomalous data collection or studies of samples with large unit-cell dimensions. The beamline has been designed in a modular manner, with functional blocks installed on common granite structures (Fig. 1 ▸). Going from the photon source down to the detector, the beam originates from an *in vacuum* U20 undulator, and is then modulated through an Si(111) channel-cut monochromator and a pair of Kirkpatrick–Baez (KB) focusing mirrors (Table 1 ▸). Along its path, the X-ray beam is shaped by four pairs of slits, two diaphragms and a pinhole. The sample holders are mounted on a three-circle goniometer, and data are recorded on a fast and large surface area X-ray detector. For the integrity of the beamline, great efforts were made towards the beam stability and the implementation of diagnostics along the line.

### 2.1. Source   

PROXIMA-1 is installed on a short straight section. The X-ray photons emerge from a 20 mm-period *in vacuum* undulator (Marcouille *et al.*, 2011 ▸), which operates at a minimum gap of 5.5 mm while taking advantage of the odd harmonics for accessing the range of energies going from 5 keV to 20 keV (Fig. S1 of the supporting information). The beam is defined by a front-end beam-defining mask with an opening of 0.47 mrad (vertical) × 0.27 mrad (horizontal). In order to enhance beam stability and quality, the undulator is ‘tapered’ (Reiser *et al.*, 2002 ▸) to allow for correction of the resulting decrease of the peak intensity height and the broadening of the harmonic. By tapering the undulator, both the beam energy loss due to spontaneous undulator radiation and the energy chirp in the electron beam are compensated, resulting in an increase of radiation power while preserving resonance conditions (Kroll *et al.*, 1981 ▸). Typically, harmonic bandwidth and intensity measurements using the taper mode of the undulator showed a reduction by 10 eV (∼12%) of the bandwidth of the 7th harmonic, coupled with an increase of ∼20% in intensity. Systematic measurements of these values on different harmonics and with various taper values validated an optimum setup that uses a taper of 30 µm (Fig. S2).

### 2.2. Optics   

The X-ray energy can be selected via a liquid-nitro­gen-cooled Si(111) channel-cut monocrystal monochromator, the first optical element seen by the beam. Careful polishing of the crystal helped to reduce (after focusing) the vertical size of the X-ray beam at the sample position to 20 µm. Fine-tuning of the energy coming out of the monochromator is performed by a piezo-based pitch stage acting directly on the crystal. A pair of first-generation KB mirrors was installed early on as the focusing optics. The quality of the mirrors is far from recent standards based on the latest polishing technologies; however, the studies approached at PROXIMA-1 (*e.g.* large-lattice macromolecular crystals) did not justify providing a micro-focused X-ray beam, and more efforts were made towards minimizing noise sources such as diffusion rather than beam size. Passing through the KB mirrors, the monochromatic X-ray beam is focused on the surface of the downstream X-ray detector when placed at the most commonly used distance from the sample position (typically at an outer shell resolution equal to 2 Å), which benefits the diffraction studies of large-unit-cell crystals. Standard divergences of 0.476 mrad × 0.259 mrad in both the horizontal and the vertical directions are recorded. When coupled with downstream slits and a diaphragm, the X-ray beam size on the sample becomes 40 µm × 20 µm (FWHM) at an energy of 12.67 keV, adopts a perfect Gaussian shape and introduces 2 × 10^11^ photons s^−1^ (at 500 mA ring current) into this area when using 100% of the incoming beam (inlet in Fig. 1 ▸). Energies ranging from 6.5 keV to 15 keV are used in the standard setup, with 12.67 keV set as the reference energy.

### 2.3. Beam diagnostics   

The implementation of a series of beam positioning monitors (BPM) and conditioning devices enables steering of the beam in addition to monitoring and maintaining quality and stability. One BPM is installed after each optical element and invading equipment (Fig. 1 ▸). The position of the beam is reflected by an electronic grade single-crystal chemical-vapour-deposition (CVD) diamond X-ray BPM (Desjardins *et al.*, 2013 ▸) located downstream of the sample slits and upstream of the fast shutter. The intensity of the X-rays and the flux deposited on the sample are measured on a duo-lateral position-sensitive detector (PSD; Desjardins *et al.*, 2018 ▸). An additional placeholder for diagnostic tools is located downstream of the mirror box (Fig. 1 ▸); it includes a series of invading elements classically used for shaping the beam and eventually modifying its transmission.

### 2.4. Goniometry and sample environment   

**Figure 2:**
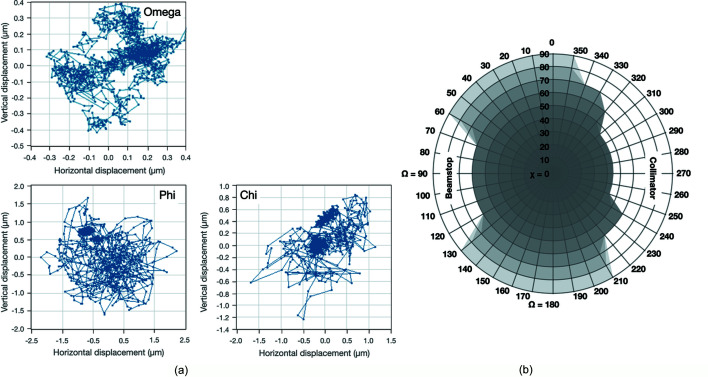
Implementation of the SmarGon within the PROXIMA-1 sample environment. (*a*) Sphere of confusion measurements for the Omega, Phi and Chi rotation axes. Each dot on the graphs corresponds to the lateral displacement of a ceramic sphere used within a calibration kit (SmarAct GmbH), measuring the deviation from an ideal rotation. (*b*) Sphere of collision at the sample environment, initially obtained by calculations over CAD-drawings and refined by visual inspection. The grey surfaces correspond to the accessible areas without the risk of hardware collision for the sample with beamline equipment when rotating the Omega (Ω) axis at different values of Chi (χ).

Since the opening of the beamline, great efforts have been made to improve sample handling, requiring numerous phases of reconstruction and accommodation of adapted instrumentation. The latest implementation is reported here as it represents the most reliable and stable setup to date. The goniometer installed at PROXIMA-1 belongs to the SmarGon series (SmarAct GmbH), adapted to the hardware requirements of the beamline. The SmarGon is a pseudo-chi three-rotation axis goniometer that provides sphere of confusion values in the sub-micrometre range for Omega (free-rotation), and below 2 µm for Phi (free-rotation) and Chi (rotation over 90°) [Fig. 2 ▸(*a*)]. Within the sphere of collisions [Fig. 2 ▸(*b*)], the goniometer provides access to a large rotation angle around the Chi-rotation axis, going up to 45° in Chi. There are various advantages emanating from the Chi-geometry for fully recording diffraction data, better orienting the crystal when recording anomalous data, or spreading the radiation dose over the entire crystal, as already reported elsewhere (Weinert *et al.*, 2015 ▸; Schiltz & Bricogne, 2008 ▸; Zeldin *et al.*, 2013 ▸). Due to its ease in use, where data collection strategies using a combination of Omega, Chi and Phi orientations were common practices for anomalous data recording at PROXIMA-1, recording full Omega-rotation data on a single crystal with different Chi-orientations has become even more popular in the user community.

**Figure 3:**
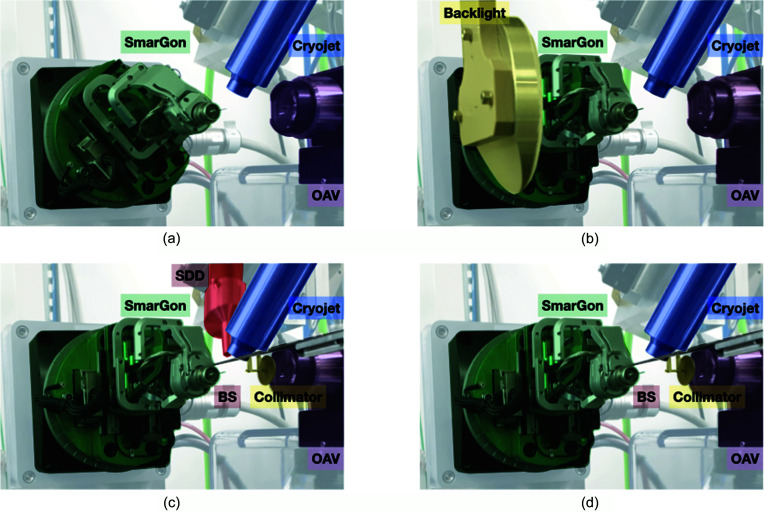
Close-up view of the sample environment in the various experimental setups, with (*a*) the sample exchange, (*b*) the visual centring, (*c*) the X-ray fluorescence and (*d*) the data collection modes. OAV, on-axis visualization camera; SDD, silicon drift detector; BS, beam stopper.

Once mounted on the goniometer head, the samples are centred to the X-ray intersection point by coupling an on-axis visualization system (OAV, Arinax Scientific Instrumentation) with a back-illumination light (Fig. 3 ▸). During data collection, the diffusion noise caused by upstream elements is removed by a pinhole mounted at the end of a hollow capillary positioned between the OAV and the sample. Downstream of the sample, a direct-beam stopper is mounted on a stage for adapting its distance from the sample to allow recording of very low-resolution Bragg reflections. The beamline is equipped with a silicon drift detector (KETEK GmbH) to perform X-ray spectroscopy measurements, confirm the atomic composition of the crystals and perform anomalous dispersion experiments. The crystals are kept under a nitro­gen vapour stream by a dedicated cryostream (700 series, Oxford Cryosystems), classically set at temperatures of 100 K. The cryogenic nozzle is mounted on a motorized stage for extraction to facilitate eventual room-temperature experiments.

### 2.5. Detector   

Following the evolution of the experimental setup, PROXIMA-1 has employed three-types of X-ray detectors. After the early implementation of a Q315r CCD camera (ADSC), the beamline operated for seven years with a PILATUS-6M X-ray detector (DECTRIS Ltd), then was later replaced in 2018 by an EigerX-16M (DECTRIS Ltd). The EigerX-16M presents smaller pixels compared with the PILATUS-6M, distributed over a smaller sensitive surface, and, considering the shorter deadtime, permits collection of very fine slicing data (Johnson *et al.*, 2014 ▸; Casanas *et al.*, 2016 ▸). While the PILATUS-6M was used at a maximum frame rate of 20 Hz, the EigerX-16M routinely records at 100 Hz on PROXIMA-1, with no major effect on the quality of the data; typical exposure time per frame at the beamline is 0.01 s over an oscillation angle of 0.1°. The installation of the detector allows access to new trends in data collection recording, such as line and grid scans, X-ray centering of the crystalline samples or *in situ* data diffraction. The detector is placed on a motorized stage to change its distance from the sample position, covering a very large set of resolutions, going from 0.75 Å at the edge of the detector when positioned at 96 mm and for 15 keV, down to 2200 Å for a sample-to-detector distance of 1550 mm, with the direct-beam stopper located 40 mm from the sample at an energy of 12.67 keV.

### 2.6. Sample changer   

A first-generation CATS sample changer (IRELEC ALCEN) has been installed on the beamline for handling samples either kept at room temperature or preserved under cryo-conditions. The reliable operation of the robot benefited from specific implementations within the sample environment in order to minimize risks for hardware collisions, and to ensure the safest possible handling. Thus, the goniometer head and the cryo-tongue of the robot were designed together for dry-mounting of the samples. The proper positioning of the instruments for a safe approach of the robot arm is confirmed by interferometry. The same interferometers are also used to confirm the proper loading of the samples on the goniometer head. The CATS itself is equipped with a current variations based collision detection, preventing the handling of mis­positioned samples, a potential cause of hardware collisions. The later implementation has been key to reduce the sample loss to negligible when handled by the robot. PROXIMA-1 allows for medium throughput experiments, with a sample exchange cycle of 40 s and with a Dewar storage that has a capacity for three Universal V1-Puck (Unipuck) cassettes, corresponding to 48 samples or 4 h experiments on average.

### 2.7. Software for beamline control, data processing and remote access   

The low-level software of the beamline has been developed in a combination of *Java Interactive Software Visualization Environment* (*Jive*; Cattaneo *et al.*, 2004 ▸) and *Python* devices. Communication between the various devices and device servers takes advantage of the *Tango Controls* protocols (https://www.tango-controls.org/). Users of PROXIMA-1 can prepare their experiments with the *ISPyB* management system (Delagenière *et al.*, 2011 ▸) and control the beamline through the *MXCuBE* graphical user interface (Gabadinho *et al.*, 2010 ▸). SOLEIL is part of the *MXCuBE* consortium (Oscarsson *et al.*, 2019 ▸), allowing users at PROXIMA-1 to benefit from the same graphical interface as in most of the MX experimental stations in Europe. In terms of beamline developments, *MXCuBE* is specifically adapted to the local equipment for controlling the instrument, with methodologies specifically developed for optimized experiments (*MXCuBE* version 2 Qt5, SOLEIL PROXIMA-1 specifics). Remote access to the beamline operation makes use of *NoMachine* (NX Technology), allowing users geographically located outside of Synchrotron SOLEIL to perform their experiments. All recorded data are processed on a 288-core computing cluster with a 30 TB SSD-RAIDS for short-term storage and 0.5 PB RAID6 for longer-term storage. Strategy calculations and data processing are typically performed via the *xdsme* (https://github.com/legrandp/xdsme) data-processing scripts based on global crystallographic packages, among which are *XDS* (Kabsch, 2010 ▸), *Pointless* (Evans, 2006 ▸), *CCP4* (Winn *et al.*, 2011 ▸) and *XDSSTAT* (Diederichs, 2006 ▸). In parallel, automated data processing can be optionally set within *MXCuBE*, which will run clever *xdsme* scripts and report processed data within the *ISPyB* web interface.

## 3. Ancillary facilities   

Users applying for beam time at Synchrotron SOLEIL have the possibility to access support laboratories, including wet laboratories dedicated to biology. Additionally, PROXIMA-1 users have access to a crystallization and observation room, where samples can be prepared and cryogenically stored prior to the experiments. A shipping dewar storage room is also available for long-term storage of samples when scheduling remote-access experiments. Finally, a computing room is available for users willing to take advantage of the computing cluster available on the beamline, also accessible from remote places over the period of the scheduled beam time.

## 4. Facility access   

Beam time at Synchrotron SOLEIL is distributed such that at least 75% is devoted to general user programs. At PROXIMA-1, up to 20% of the available beam time is reserved for commercial usage, 10% is used for calls of rapid access and commissioning of the beamline, and the rest is devoted to experiments by academic users. There are two calls for proposals per year and a peer-review committee reviews proposals based on scientific merit and beamline suitability. For MX experiments, users have the option to submit block allocation group (BAG) or standard proposals. Approved standard proposals are valid for 6 months, whereas BAGs extend to 2 years. The final scheduling of the assigned beam time is performed by the beamline staff on the basis of 8 h shifts. Synchrotron SOLEIL provides three modes of access to the beamline, including physical presence of the users, remote access experiments via secured protocols and mail-in data collection for industrial users interested in taking advantage of dedicated PROXIMA-1 staff to perform data collection and processing.

## 5. Highlights   

Between 2008 and the end of 2020, research output on PROXIMA-1 has assisted in producing more than 1000 peer-reviewed papers and reached an average of 100 scientific publications per year in the last 7 years of operation. In an attempt to provide some detail on the main scientific trends performed on the beamline, four articles are hereafter highlighted.

### 5.1. Anomalous phasing   

Anomalous phasing is one of the key expertise of PROXIMA-1. Highly standardized at all synchrotron sites with access to specialized instruments, it remains challenging when not properly applied or when the quality of the recorded data is not satisfactory. Trowitzsch *et al.* (2015 ▸) showed that the high quality of the data recorded was mandatory for the structure determination by S-SAD of one TATA-binding protein associated factor complex (TAF8-10). The experimental results provided key insights towards the unravelling of a stepwise assembly pathway of preformed cytoplasmic submodules that control direct gene transcription and are regulated by nuclear import.

### 5.2. PROXIMA-1 data as part of multi-modal projects   

Synchrotron SOLEIL covers a large energy spectrum, allowing various techniques to be integrated and also permits tackling specific issues from various approaches. The Biology and Health scientific section regroups most of the beamlines that provide options within the spectral domains adapted to biological samples. Considering these options, results obtained at PROXIMA-1 are often coupled with data recorded on other beamlines. For instance, in the work by Poonsiri *et al.* (2018 ▸), structures of the nonstructural protein 1 (NS1) from flavivirus solved at PROXIMA-1 were compared with small-angle scattering data recorded at the SAXS beamline SWING, a comparison that emphasized a number of differences in NS1 proteins and contributed to insightful information involving the relationships between the NS1 structures and their pathogenic functions towards the development of better diagnostics.

### 5.3. Complementarity to cryo-electron microscopy   

Structural biology recently witnessed a major revolution to what is being called ‘integrated biology’. In integrated biology, techniques are combined so as to fulfil the same goal, generally to gain further insights into complex mechanisms or architectural macromolecular arrangements. In this context, the study of large objects by X-ray diffraction has evolved by integrating cryo-electron microscopy (cryoEM) studies up to medium-to-high resolution, coupled with higher-resolution MX studies. An early inspiring work from Procházková *et al.* (2018 ▸) combined cryoEM results to crystallographic data, with the overall idea to push for a better understanding of complicated biological mechanisms at higher resolution. In their studies, the authors highlighted differences in the shapes of various virions and explained the pH-dependent infectious mechanisms of honeybee Sacbrood viruses. The virion crystal lattices in these studies presented large unit cell values of above 480 Å, 360 Å and 330 Å for the *a*, *b* and *c* axes, respectively, highlighting the perfect match of these studies with the specificities at PROXIMA-1 to work on large macromolecular assemblies.

### 5.4. Pharmaceutical investigations   

Short of welcoming industrial users, PROXIMA-1 has witnessed industrial-driven scientific discoveries that have proven to be strategic in the development of effective drugs. Kotschy *et al.* (2016 ▸) highlighted the importance of the pro-survival protein myeloid cell leukaemia 1 (MCL1) as a target for small molecules amenable for clinical testing to potentially kill MCL1-dependent cancer cells by activating the BAX/BAK-dependent mitochondrial apoptotic pathway. This research is an example of key structural discoveries with deep societal implications, resulting from an industrial project and investigations.

## 6. Discussion and conclusions   

In summary, the PROXIMA-1 beamline for macromolecular crystallography was developed at Synchrotron SOLEIL and has successfully served the crystallographic user community for over 12 years. The beamline is adapted for large cell dimensions, anomalous diffraction and complex data collection strategies. Together with a high-speed X-ray detector, the three-rotation axis goniometer installed on the beamline is optimized for fast data collection of highly redundant data, taking advantage of the micrometre-size sphere-of-confusion in the three-rotation axis. The size of the X-ray beam was optimized for 100 µm-long crystals, fully complementary to the micro-focus MX beamline at Synchrotron SOLEIL. The quality of the recorded data and stability of the beamline has allowed the release of over 1400 entries in the Protein Data Bank (as of December 2020), leading to the publication of a significative number of scientific reports.

## Supplementary Material

Supporting Figues S1 and S2. DOI: 10.1107/S1600577521002605/ay5573sup1.pdf

## Figures and Tables

**Table 1:** PROXIMA-1 beamline details

Beamline	PROXIMA-1
Source type	U20 *in vacuum* undulator
Monochromator	Si(111) single-crystal channel-cut
Mirrors	Vertical and horizontal KB (Rh-coated glass) at a grazing angle of 4.1 mrad
Energy range (keV)	6.5–15
Wavelength range (A)	0.82–1.9
Beam size, uncollimated (µm^2^)	120 (H) × 20 (V), FWHM
Beam size, collimated (µm^2^)	40 (H) × 20 (V), FWHM
Flux, uncollimated (photons s^−1^)	3.8 × 10^11^ at 12.67 keV and a ring current of 500 mA
Flux, collimated (photons s^−1^)	2.1 × 10^11^ at 12.67 keV and a ring current of 500 mA
Goniometer	Three-rotation axis SmarGon, air-bearing
Cryo capability	Oxford Cryosystems 700-series
Sample exchange robot	CATS, 3 Unipucks, 48 samples
Detector type, model	Single-photon-counting pixel-array EigerX-16M
Classical frame rate (Hz)	100
