# Supplementary material for: PROXIMA-1 beamline for macromolecular crystallography measurements at Synchrotron SOLEIL
Source: J Synchrotron Radiat. 2021 Apr 29;28(Pt 3):970–6. doi: 10.1107/S1600577521002605 (PMC8127379; doi:10.1107/S1600577521002605)
Supplement: Supplementary file 1 [file s-28-00970-sup1.pdf]

JOURNAL OF SYNCHROTRON RADIATION

Volume 28 (2021)

Supporting information for article:

PROXIMA-1 beamline for Macromolecular Crystallography measurements at Synchrotron SOLEIL

Leonard M.G. Chavas, Patrick Gourhant, Beatriz G. Guimaraes, Tatiana Isabet, Pierre Legrand, Robin Lener, Pierre Montaville, Serena Sirigu and Andrew Thompson

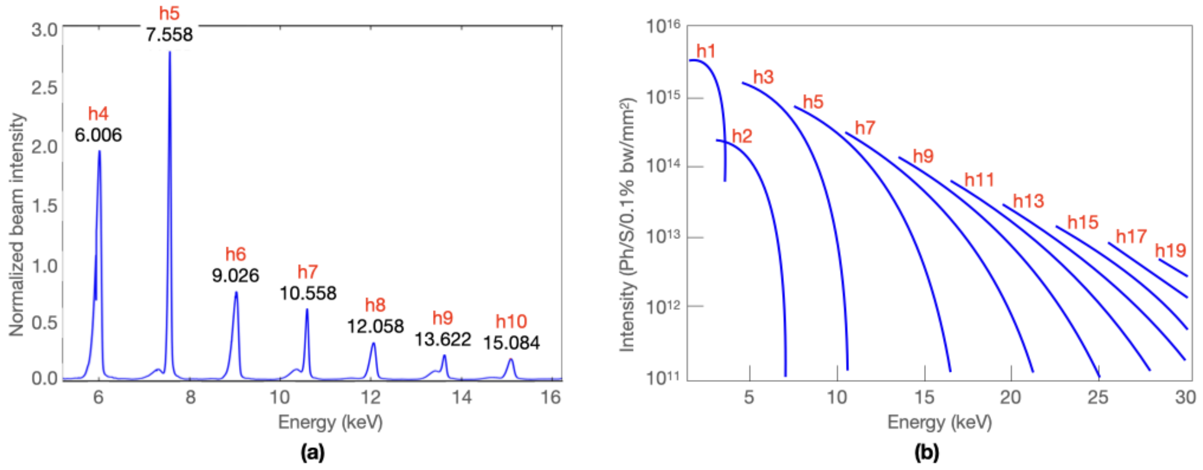

Figure S1 **(a)** Measured distribution of the available harmonics (numbered in red) coming out of the U20 undulator for a fixed closed gap of 5.5 mm at PROXIMA-1. On the top of each harmonic is also indicated the energy value at the peak. **(b)** Calculated tuning curve representing the beam flux depending on the photon energy for the odd harmonics (mainly 3rd, 5th, 7th and 9th harmonics, respectively).

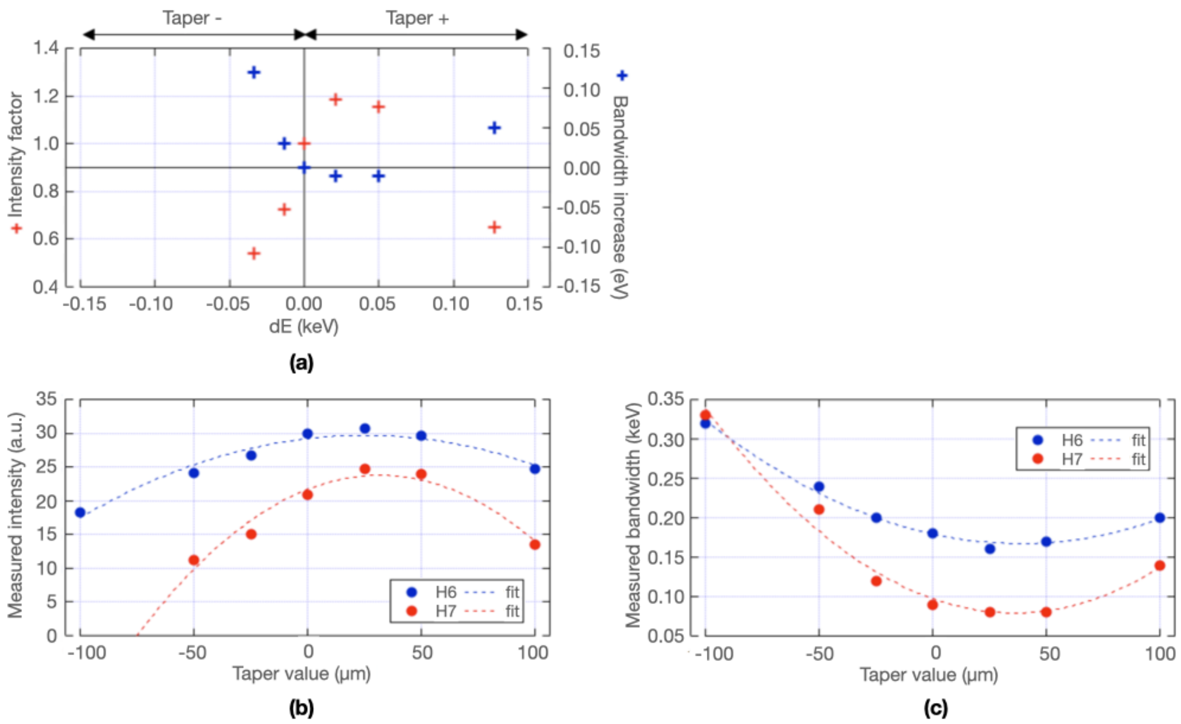

Figure S2 Improvement of the quality of the X-ray beam at PROXIMA-1 with the implementation of the taper mode on the undulator. **(a)** Gain in intensity (red) and variations of the bandwidth (blue) for the 7th harmonic depending on a small shift in energy (*dE*) caused by negative and positive tapering (*Taper* – and *Taper* +, respectively) and in comparison, with the situation where no taper-correction is

applied (*dE = 0.00*). Variations in intensities **(b)** and in bandwidth **(c)** for the 6th (blue) and 7th (red) harmonics depending on the variation in taper values.
